# Machine-Learning Assisted Discrimination of Precancerous and Cancerous from Healthy Oral Tissue Based on Multispectral Autofluorescence Lifetime Imaging Endoscopy

**DOI:** 10.3390/cancers13194751

**Published:** 2021-09-23

**Authors:** Elvis Duran-Sierra, Shuna Cheng, Rodrigo Cuenca, Beena Ahmed, Jim Ji, Vladislav V. Yakovlev, Mathias Martinez, Moustafa Al-Khalil, Hussain Al-Enazi, Yi-Shing Lisa Cheng, John Wright, Carlos Busso, Javier A. Jo

**Affiliations:** 1 Department of Biomedical Engineering, Texas A&M University, College Station, TX 77843, USA; eduran@tamu.edu (E.D.-S.); csncbmp@hotmail.com (S.C.); yakovlev@tamu.edu (V.V.Y.); 2 School of Electrical and Computer Engineering, University of Oklahoma, Norman, OK 73019, USA; rodrigo.cuenca@ou.edu; 3 School of Electrical Engineering and Telecommunications, University of New South Wales, Sydney 2052, Australia; beena.ahmed@unsw.edu.au; 4 Department of Electrical and Computer Engineering, Texas A&M University at Qatar, Doha 23874, Qatar; jim.ji@qatar.tamu.edu; 5 Department of Cranio-Maxillofacial Surgery, Hamad Medical Corporation, Doha 3050, Qatar; mcoronel@hamad.qa (M.M.); malkhalil@hamad.qa (M.A.-K.); 6 Department of Otorhinolaryngology Head and Neck Surgery, Hamad Medical Corporation, Doha 3050, Qatar; halenazi@hamad.qa; 7 College of Dentistry, Texas A&M University, Dallas, TX 75202, USA; YCheng@tamhsc.edu (Y.-S.L.C.); JWright@tamhsc.edu (J.W.); 8 School of Electrical and Computer Engineering, The University of Texas at Dallas, Dallas, TX 75080, USA; busso@utdallas.edu

**Keywords:** oral cancer and dysplasia, positive surgical margin detection, multispectral autofluorescence lifetime imaging (maFLIM), autofluorescence biomarkers, machine learning

## Abstract

**Simple Summary:**

Complete resection of dysplastic and malignant tissue improves overall survival and delays cancer recurrence in oral cancer patients; however, intraoperative surgical margin assessment is limited to visual inspection and palpation, making it difficult to achieve total resection. There is currently no tool capable of providing real-time, accurate, and continuous margin-assessment guidance during oral cancer resection surgery. Multispectral autofluorescence lifetime imaging (maFLIM) is a label-free imaging modality that enables quantifying a plurality of metabolic and compositional autofluorescence biomarkers of oral dysplasia and cancer. We have developed and validated a machine-learning assisted computer aided detection (CAD) system for automated discrimination of dysplastic and cancerous from healthy oral tissue based on in vivo widefield maFLIM endoscopy data. This CAD system can be potentially embedded into maFLIM endoscopes to enable continuous in situ detection of positive margins during oral cancer resection surgery, thus facilitating maximal tumor resection and improving surgical outcomes for oral cancer patients.

**Abstract:**

Multispectral autofluorescence lifetime imaging (maFLIM) can be used to clinically image a plurality of metabolic and biochemical autofluorescence biomarkers of oral epithelial dysplasia and cancer. This study tested the hypothesis that maFLIM-derived autofluorescence biomarkers can be used in machine-learning (ML) models to discriminate dysplastic and cancerous from healthy oral tissue. Clinical widefield maFLIM endoscopy imaging of cancerous and dysplastic oral lesions was performed at two clinical centers. Endoscopic maFLIM images from 34 patients acquired at one of the clinical centers were used to optimize ML models for automated discrimination of dysplastic and cancerous from healthy oral tissue. A computer-aided detection system was developed and applied to a set of endoscopic maFLIM images from 23 patients acquired at the other clinical center, and its performance was quantified in terms of the area under the receiver operating characteristic curve (ROC-AUC). Discrimination of dysplastic and cancerous from healthy oral tissue was achieved with an ROC-AUC of 0.81. This study demonstrates the capabilities of widefield maFLIM endoscopy to clinically image autofluorescence biomarkers that can be used in ML models to discriminate dysplastic and cancerous from healthy oral tissue. Widefield maFLIM endoscopy thus holds potential for automated in situ detection of oral dysplasia and cancer.

## 1. Introduction

Oral cancer is a significant global health threat with ~355,000 cases and over 177,000 deaths each year, and one of the lowest five-year survival rates (~50%) among the major cancer types [1]. This threat is greatly attributed to the difficulty in capturing all the cancer at treatment. Oral cancer treatment is primarily surgical with the aim of achieving complete tumor resection without leaving behind residual disease [2]. Positive surgical margins are associated with significant increase in recurrence rate and decrease in survival rate [3]; unfortunately, the rate of oral cancer positive surgical margins can be as high as 40% [4]. Intraoperative oral cancer surgical margin assessment is limited to visual inspection and palpation. When available, histopathological evaluation of intraoperative frozen sections is also used [2,5], but it suffers from low sensitivity (as low as 15%) [6,7], and its positive impact in clinical outcomes is unclear [8,9].

Preoperative positron emission tomography (PET), computed tomography (CT), and magnetic resonance imaging (MRI) are routinely used in oral cancer staging and surgical planning, but they are not useful for intraoperative surgical margin assessment [10,11]. Optical imaging modalities, which can provide simultaneous structural, functional, and biochemical tissue characterization across multiple scales, are ideal for intraoperative surgical margin assessment. Grillone et al. performed an in vivo study on 34 patients, in which elastic scattering spectroscopy (ESS) and a machine learning diagnostic algorithm were used to distinguish healthy from abnormal (mild/moderate/severe dysplasia, carcinoma in situ, and invasive cancer) oral tissue, with sensitivity ranging from 84% to 100% and specificity ranging from 71% to 89%, depending on how the cutoff between healthy and abnormal tissue was defined (i.e., mild, moderate, or severe dysplasia) [12]. In an ex vivo study, Hamdoon et al. used optical coherence tomography (OCT) to scan tumor margins from 28 oral squamous cell carcinoma (OSCC) patients following resection, and they differentiated tumor-free from tumor-involved margins with levels of sensitivity and specificity of 81.5% and 87%, respectively [13]. Jeng et al. used Raman spectroscopy (RS) in an ex vivo study to image 44 tumor and 36 healthy oral tissue samples from patients and implemented a quadratic discriminant analysis (QDA) classifier to discriminate tumor from healthy oral tissue, resulting in levels of sensitivity and specificity of ~91% and ~83%, respectively [14]. Halicek et al. performed ex vivo hyperspectral imaging (HSI) on 20 patients and discriminated SCC margins from healthy oral tissue with 84% sensitivity and 74% specificity using a convolutional neural network classifier [15]. Nayak et al. used autofluorescence spectroscopy (AFS) and an artificial neural network to classify healthy (*n* = 40) vs. premalignant (*n* = 6) and malignant (*n* = 37) oral tissue biopsies from patients and reported levels of sensitivity and specificity of 96.5% and 100%, respectively [16]. Unfortunately, none of these technologies have been yet translated to the operating room; thus, intraoperative image-guiding technologies that will facilitate complete oral tumor resection are still urgently needed.

Two mitochondrial metabolic coenzymes, the reduced-form nicotinamide adenine dinucleotide (NADH) and flavin adenine dinucleotide (FAD), are used in multiple metabolic processes, including glycolysis and oxidative phosphorylation, and are the main endogenous fluorophores in the oral epithelial layer [17,18]. Increased cellular metabolic activity, a hallmark of malignant epithelial cells, can be quantified by imaging the oral tissue autofluorescence originated from the metabolic cofactors NADH and FAD [17,19,20]. We have recently demonstrated clinical label-free metabolic imaging of oral epithelial cancer based on multispectral autofluorescence lifetime imaging (maFLIM) endoscopy in patients presenting oral malignant lesions and reported several autofluorescence biomarkers of oral epithelial cancer [20].

These capabilities and its relatively inexpensive implementation cost make maFLIM a promising imaging modality to enable continuous real-time margin-assessment guidance during oral cancer resection surgery. However, for maFLIM to become an impactful image-guided tool for oral cancer resection surgery, computer-aided detection (CAD) systems are needed to enable in situ intraoperative, automated, objective, and accurate discrimination and visualization of dysplastic and cancerous vs. healthy oral tissue during tumor resection surgery. In this study, we report what is, to the best of our knowledge, the first independently validated CAD system for automated clinical detection of dysplastic and cancerous from healthy oral tissue based on in vivo widefield multispectral autofluorescence lifetime imaging endoscopy.

## 2. Materials and Methods

**Figure 1:**
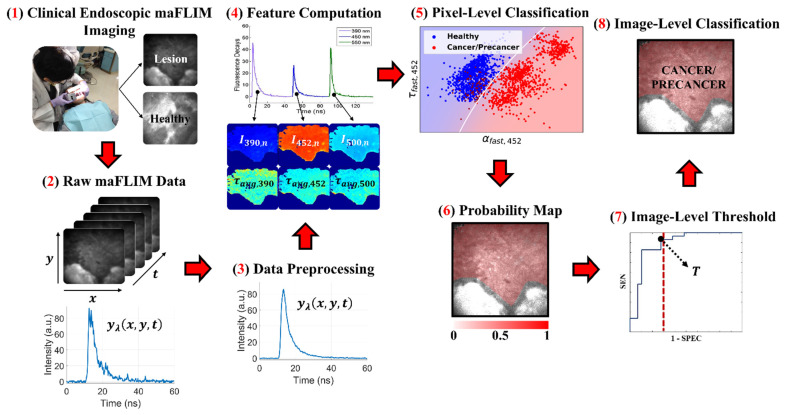
Summary of the methods used in this study. (**1**) In vivo clinical maFLIM images of both the lesions and healthy tissue regions from oral cancer patients were acquired. (**2**,**3**) Raw maFLIM data were preprocessed to increase the signal quality. (**4**) Autofluorescence spectral and time-resolved maFLIM features were computed per pixel. (**5**) Models for the classification of precancer/cancer vs. healthy oral tissue at the pixel level were trained. (**6**) Pixel-level classification results in a posterior probability map for each imaged oral tissue region. (**7**) An image-level score was computed from the posterior probability map, and a threshold (T) on this score was optimized. (**8**) The image-level score threshold was applied to classify the whole image as either precancer/cancer or healthy. Note. Modified from “Clinical label-free biochemical and metabolic fluorescence lifetime endoscopic imaging of precancerous and cancerous oral lesions,” by Duran-Sierra, E.; Cheng, S.; Cuenca-Martinez, R.; Malik, B.; Maitland, K.C.; Lisa Cheng, Y.S.; Wright, J.; Ahmed, B.; Ji, J.; Martinez, M.; et al., 2020, *Oral Oncol*, p. 2, doi:10.1016/j.oraloncology.2020.104635 [20].

A summary of the methods applied in this study, from the maFLIM data acquisition to the final classification output, is shown in Figure 1 and described in detail in the following sections.

### 2.1. Clinical Endoscopic maFLIM Imaging of Oral Lesions

#### 2.1.1. Training Set

In vivo clinical maFLIM endoscopy images of dysplastic and cancerous oral lesions were acquired following an imaging protocol approved by the Institutional Review Board at Hamad Medical Corporation (Doha, Qatar). In this study, 34 patients scheduled for tissue biopsy examination of suspicious oral epithelial precancerous or cancerous lesions were recruited. Following clinical examination of the patient’s oral cavity by an experienced head and neck surgeon (M.M., M.A.K., H.A.E), maFLIM endoscopy images were acquired from both the suspicious oral lesion and a clinically healthy-appearing area in the corresponding contralateral anatomical side, using a maFLIM endoscope previously reported in Cheng et al. [21]. Tissue autofluorescence excited with a pulsed laser (355 nm, 1 ns pulse width, ~1 μJ/pulse at the tissue) was imaged at the emission spectral bands of 390 ± 20 nm, 452 ± 22.5 nm, and >500 nm, which were selected to preferentially image collagen, NADH, and FAD autofluorescence, respectively. The total energy deposited into the patient’s oral mucosa (2.8 mJ) was set to at least an order of magnitude lower than the maximum permissible exposure (MPE = 29.8 mJ) provided by the American National Standards Institute (ANSI) [22]. Each maFLIM endoscopy image was acquired with a circular field-of-view (FOV) of ~11 mm in diameter, lateral resolution of ~100 μm, and total acquisition time of <3 s. The time-resolved autofluorescence intensity signal measured at each pixel was digitally sampled at 4 GS/s. After acquiring the maFLIM endoscopy images from the oral lesion biopsy region and corresponding contralateral healthy area determined by the surgeon, the tissue biopsy examination procedure was performed following standard clinical protocols. All biopsies performed were incisional taken from the center of the lesion and of different sizes based on the type and extension of lesion examined. The imaged clinically healthy-appearing areas on the contralateral side of the lesions were not biopsied. Each imaged lesion was then annotated based on the corresponding tissue biopsy histopathological diagnosis (gold standard). The distribution of the 34 imaged oral lesions based on both anatomical location and histopathological diagnosis is provided in Table 1, and the demographic information of the 34 imaged patients is summarized in Table 2.

#### 2.1.2. Testing Set

Additional in vivo clinical maFLIM endoscopy images of oral lesions and healthy oral tissue were acquired from 23 patients from the Texas A&M University College of Dentistry (Dallas, TX, USA), following a similar imaging protocol approved by the Institutional Review Board at Texas A&M University (College Station, TX, USA). The maFLIM endoscopy system used to image this cohort of patients had the same characteristics as the one previously described and reported in Cheng et al. [21], except for the sampling rate, which was 6.25 GS/s. The distribution of these additional 23 imaged oral lesions based in both anatomical location and histopathological diagnosis is also provided in Table 1, and the demographic information of the 23 imaged patients is summarized in Table 2.

### 2.2. maFLIM Feature Extraction

In order to generate a maFLIM feature pool, the endoscopic maFLIM images were processed as follows. The maFLIM data are composed of fluorescence intensity temporal decay signals, $y_{\lambda }\left(x,y,\;t\right),$ measured at each emission spectral band (λ) and at each spatial location or image pixel (x,y). Each maFLIM dataset was first preprocessed as follows. First, offset and background subtraction was applied to the temporal signal at each pixel of the maFLIM image. Second, pixels presenting temporal signal saturation were detected by setting a threshold on the maximum signal amplitude, and masked. Third, spatial averaging (order 5 × 5) was applied to increase the temporal signal-to-noise ratio (SNR) at each spatial location. Fourth, pixel masking based on SNR was also performed with an SNR threshold value of 15 decibels. Finally, additional pixels were manually masked from regions where tooth areas were observed in the intensity images.

After data preprocessing, absolute and normalized multispectral fluorescence intensity values were computed for each pixel as follows. The absolute multispectral fluorescence intensity $I_{\lambda }\left(x,y\right)$ was computed by numerically integrating the fluorescence intensity temporal decay signal (Equation (1)).

(1) $$I_{\lambda }\left(x,y\right)=\int y_{\lambda }\left(x,y,t\right)dt$$

The normalized multispectral fluorescence intensity $I_{\lambda ,\;n}\left(x,y\right)$ was computed from the absolute multispectral fluorescence intensities $I_{\lambda }\left(x,y\right)$ using Equation (2).

(2) $$I_{\lambda ,n}\left(x,y\right)=\frac{I_{\lambda }\left(x,y\right)}{\sum_{\lambda }I_{\lambda }\left(x,y\right)}$$

From the multispectral absolute fluorescence intensities, six intensity ratios were computed at each spatial location. Three quantify the relative autofluorescence intensities between single spectral channels: $I_{390}\left(x,y\right)$/$I_{452}\left(x,y\right)$, $I_{390}\left(x,y\right)$/$I_{500}\left(x,y\right)$, and $I_{452}\left(x,y\right)$/$I_{500}\left(x,y\right)$; and three quantify the combined autofluorescence intensity of two spectral channels relative to the third one:$\;(I_{452}\left(x,y\right)+I_{500}\left(x,y\right))/I_{390}\left(x,y\right)$, $(I_{390}\left(x,y\right)+I_{500}\left(x,y\right))/I_{452}\left(x,y\right)$, and $(I_{390}\left(x,y\right)+I_{452}\left(x,y\right))/I_{500}\left(x,y\right)$.

In the context of time-domain maFLIM data analysis, the fluorescence decay $y_{\lambda }\left(x,y,\;t\right)$ measured at each spatial location (x,y) can be modeled as the convolution of the fluorescence impulse response (FIR) $h_{\lambda }\left(x,y,\;t\right)$ of the sample and the measured instrument response function (IRF) $u_{\lambda }\left(t\right)$ as shown in Equation (3).

(3) $$y_{\lambda }\left(x,y,t\right)=u_{\lambda }\left(t\right)\;*\;h_{\lambda }\left(x,y,t\right)\;$$

Therefore, to estimate the sample FIR $h_{\lambda }\left(x,y,\;t\right)$, the measured IRF $u_{\lambda }\left(t\right)$ needs to be temporally deconvolved from the measured fluorescence decay $y_{\lambda }\left(x,y,\;t\right)$. In this work, temporal deconvolution was performed using a nonlinear least-squares iterative reconvolution algorithm [23], in which the FIR was modeled as a multiexponential decay. The model order (number of exponential components) was determined based on the model-fitting mean-squared error (MSE); since the addition of a third component did not reduce the MSE, a bi-exponential model (order of two) was selected (Equation (4)).

(4) hλ(x,y,t)=αfast,λ(x,y)e−tτfast,λ(x,y)+αslow,λ(x,y)e−tτslow,λ(x,y)

Here, $\tau_{fast,\lambda }\left(x,y\right)$ and $\tau_{slow,\lambda }\left(x,y\right)$ represent the time-constant (lifetime) of the fast and slow decay components, respectively; while $\alpha_{fast,\lambda }\left(x,y\right)$ and $\alpha_{slow,\lambda }\left(x,y\right)$ represent the relative contribution of the fast and slow decay components, respectively. Finally, the average fluorescence lifetime ($\tau_{avg,\lambda }\left(x,y\right)$) for each pixel and emission spectral band were estimated from the FIR $h_{\lambda }\left(x,y,\;t\right)$ using Equation (5) [23]:

(5) $$\tau_{avg,\lambda }\left(x,y\right)=\frac{\int th_{\lambda }\left(x,y,t\right)dt}{\int h_{\lambda }\left(x,y,t\right)dt}$$

In summary, a total of 21 maFLIM-derived features were computed per pixel as summarized in Table 3.

### 2.3. Classification Model Optimization Using the Training Set

Four traditional ML classification models were evaluated with the computational framework depicted in Figure 1: Linear Discriminant Analysis (LDA) [24], Quadratic Discriminant Analysis (QDA) [25], linear Support Vector Machines (SVM; L2-regularization; C = 100) [26], and Logistic Regression (LOGREG) [27]. First, a trained classification model was applied at the pixel level resulting in a posterior probability map, from which an image-level score was computed consisting in the average of the squared pixel-level posterior probabilities, similar to the Brier score [28]. Then, ROC analysis was performed on the image-level scores, and an image-level score threshold was optimized by selecting the point on the ROC curve with maximum sensitivity within the (1-specificity) range of 0–30%. Finally, the whole image was classified as positive (dysplasia/cancer) if the image-level score was greater than or equal to the threshold, or as negative (healthy) otherwise.

To identify optimal classification models for each feature pool evaluated (spectral-only and time-resolved-only), the dataset of 34 multiparametric maFLIM images of oral lesions and 34 paired contralateral healthy images was analyzed following a 7-fold cross-validation strategy. The dataset consisting in a total of 68 maFLIM images was divided in seven folds, six of them containing 10 maFLIM images (5 lesion and 5 paired healthy images) each, and one containing 8 maFLIM images (4 lesion and 4 paired healthy images). At every iteration, six folds were used for training and one for validation. First, the six training folds entered a sequential forward search feature selection stage [29,30], in which feature sets containing up to three features were generated by iteratively adding one feature at a time based on the maximum receiver operating characteristic area under the curve (ROC-AUC) obtained from the training fold classification. At the end of this stage, three classification models with either one, two, or three features were identified, and their corresponding ROC-AUC values were recorded. The optimal classification model (and corresponding optimal feature set and image score threshold) was selected based on the largest ROC-AUC value. The selected optimal classification model was then applied to the validation fold. Finally, the whole process was repeated until each of the seven folds was used as the validation fold.

The discriminatory power of the spectral and time-resolved feature pools combined was also investigated through the implementation of an ensemble classifier. After the optimal classification models with spectral-only and time-resolved-only features were identified, the 7-fold cross-validation strategy was applied to optimize an ensemble classifier combining the best performing spectral-only and time-resolved-only classification models. In this cross-validation strategy, the training folds were used to train the previously identified optimal models with their three most frequent either spectral or time-resolved features, respectively, and the weighted sum of their resulting posterior probability maps was computed. No additional feature selection was performed in this process. The image level-scores were then computed from the weighted sum of the posterior probability maps and used to optimize an image-level threshold for the ensemble model. The trained ensemble classifier and optimized image-level threshold were then applied to the validation fold. The weights used to compute the sum of the two posterior probability maps were normalized as $w_{1}+w_{2}=1$ and optimized by repeating the 7-fold cross-validation process for every value of $w_{1}$ between 0 and 1 with an increment of 0.1. 

For each model, a confusion matrix was generated after completing the 7-fold cross-validation, and the resulting sensitivity, specificity, and F1-score were computed using Equations (6)–(8), respectively.

(6) $$Sensitivity=\frac{TP}{TP+FN}$$

(7) $$Specificity=\frac{TN}{TN+FP}$$

(8) $$F1=\frac{TP}{TP+\frac{1}{2}\left(FP+FN\right)}$$

where *TP*, *FN*, *TN*, and *FP* represent the number of true positives, false negatives, true negatives, and false positives, respectively.

## 3. Results

### 3.1. Classification Model Optimization Using the Training Set

**Figure 2:**
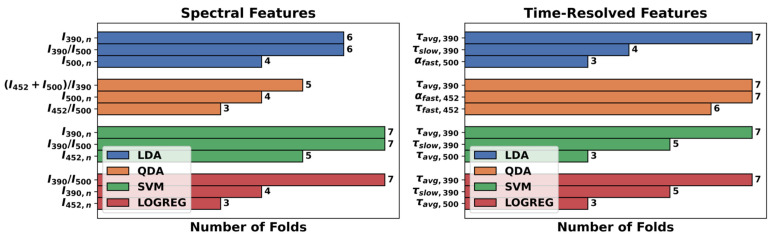
Frequency of the top three maFLIM features selected for each feature pool and classification model.

Table 4 summarizes the results of the 7-fold-cross-validation strategy applied to each combination of maFLIM feature pools (spectral-only vs. time-resolved-only) and classification models (LDA, QDA, SVM, LOGREG). The best performing classification model using spectral-only features was SVM, while QDA was the best performing model using time-resolved-only features. It should be noticed that at each cross-validation fold, a different subset of features can be selected; thus, there is no unique optimal subset of features. To identify the most relevant features, the frequency of the three most selected maFLIM features in the seven folds for each classification model evaluated is presented in Figure 2. 

For the ensemble classifier, the SVM model was retrained with the top three spectral features ($I_{390,n}$, $I_{390}/I_{500}$, $I_{452,n}$), and the QDA model was retrained with the top three time-resolved features ($\tau_{avg,\;390}$, $\alpha_{fast,452}$, $\tau_{fast,452}$). An optimal weight of $w_{1}=0.4$ was selected, as it maximized sensitivity (Equation (6)) and F1-score (Equation (8)) without decrementing too much specificity (Equation (7)). The best performance of the SVM-QDA ensemble classifier (with $w_{1}=0.4$) and the performance of the SVM and QDA models alone retrained with their corresponding top three features are also reported in Table 4.

The confusion matrices resulting from the 7-fold cross-validation for the best performing classification models are shown in Table 5, where the image-level classification model predictions are compared against the true histopathological classification (gold standard). The ensemble SVM-QDA classification model produced the highest cross-validation sensitivity (94%), specificity (74%), and F1-score (0.85). 

### 3.2. Independent Classification Performance Quantification in the Testing Set

The identified optimal classification models (Table 4) were retrained using all the maFLIM images acquired from the patient population at Hamad Medical Corporation in Doha-Qatar (training set, *n* = 34). These classification models were then ‘locked’ and applied without any further modification to all the acquired maFLIM images from the patient population at the Texas A&M University, College of Dentistry in Dallas, TX (testing set, *n* = 23). 

**Figure 3:**
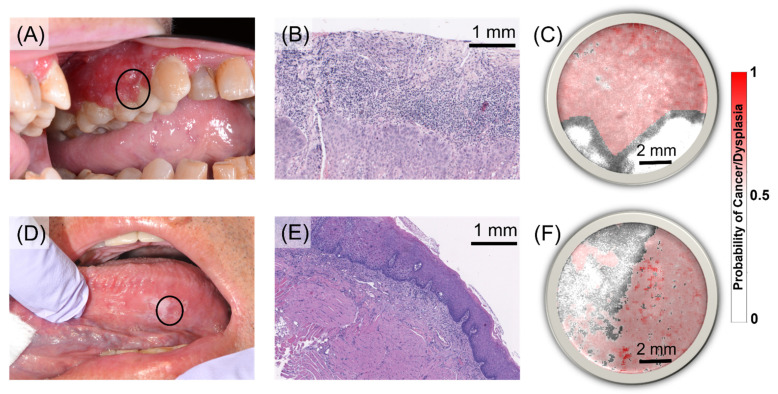
Representative imaged, diagnosed, and classified cancerous and precancerous oral lesions from the testing set. Top: (**A**) Red, inflamed lesion in left maxillary buccal gingiva (black circle indicates approximate location of the acquired maFLIM image FOV). (**B**) Histological examination of an incisional biopsy revealed moderately differentiated squamous cell carcinoma (SCC) (Scalebar: 1 mm). (**C**) Posterior probability map (red intensity scale) superposed on the total fluorescence intensity map (grey intensity scale) of the gingiva lesion obtained from the SVM-QDA ensemble classifier (Scalebar: 2 mm). Bottom: (**D**) White plaques in left lateral ventral tongue. (**E**) Histological examination of an incisional biopsy revealed mild-to-moderate epithelial dysplasia (MoD) (Scalebar: 1 mm). (**F**) Posterior probability map (red intensity scale) superposed on the total fluorescence intensity map (grey intensity scale) of the tongue lesion obtained from the SVM-QDA ensemble classifier (Scalebar: 2 mm).

Representative cases from the testing set independently classified are shown in Figure 3. The first case corresponds to a patient presenting a red, inflamed lesion of approximately 5 × 1 cm^2^ in the left maxillary gingiva (Figure 3A). Histological examination of an incisional biopsy taken from the affected gingiva revealed moderately differentiated squamous carcinoma (SCC) with a maximum depth of invasion of 2.4 mm (Figure 3B). The CAD system generates precancer/cancer posterior probability maps produced by applying the optimized classification model to each pixel of a maFLIM image. For visualization, the posterior probability map (red intensity scale) is superposed over the total fluorescence intensity map (grey intensity scale). The SVM-QDA ensemble classifier posterior probability map of the SCC lesion showed homogeneous probability values greater than 0.5 in the region corresponding to the gingiva tissue; the white areas corresponded to teeth regions (Figure 3C). 

The second case corresponds to a patient presenting scattered white plaques on the left lateral ventral tongue (Figure 3D). Histological examination of an incisional biopsy taken from the affected tongue area revealed mild-to-moderate epithelial dysplasia with overlying hyperparakeratosis (Figure 3E). The corresponding SVM-QDA ensemble classifier posterior probability map showed two regions, one with probability values greater than 0.5, and another with probability values less than 0.5 (Figure 3F). The posterior probability maps generated by the SVM-QDA ensemble classification model for all maFLIM images included in the testing set are presented in Figure S1 in the Supplemental Materials. 

**Figure 4:**
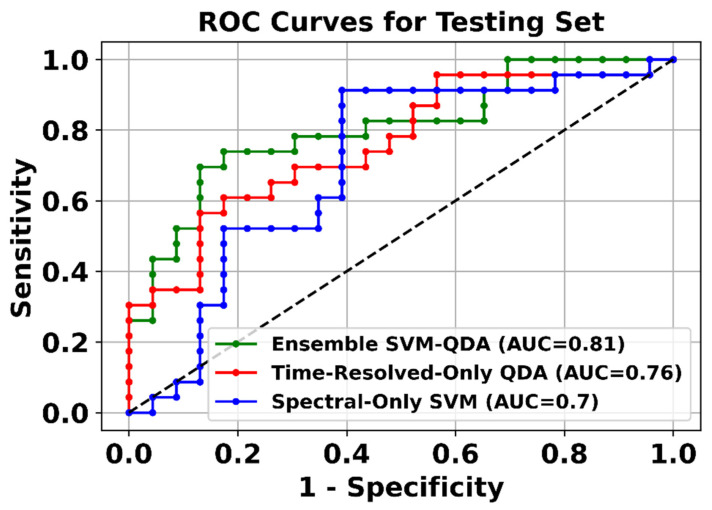
ROC curves from the complete testing set classification results for each classification model.

The classification performance of each optimal classifier was independently quantified from the testing set classification results in terms of the ROC-AUC. The ROC curves from the complete testing set classification results for each classification model are presented in Figure 4. The ensemble classifier, combining both spectral and time-resolved features, showed the highest ROC-AUC (0.81). The confusion matrices resulting from the application of the optimal classification models (SVM, QDA, and SVM-QDA ensemble) to the testing set are presented in Table S1 in the Supplemental Materials.

## 4. Discussion

In this study, clinical widefield label-free metabolic imaging of cancerous and dysplastic oral lesions was successfully performed at two clinical centers using previously developed multispectral autofluorescence lifetime imaging (maFLIM) endoscopic instruments [21]. The maFLIM metabolic images from 34 patients acquired at one of the clinical centers (Hamad Medical Corporation, Doha, Qatar) were used to optimize and train statistical classification models for automated detection of dysplastic and cancerous oral lesions. A CAD system was then developed based on the optimized classification models and applied to an independent set of maFLIM metabolic images from 23 patients acquired at the other clinical center (Texas A&M College of Dentistry, Dallas, TX, USA).

We have previously demonstrated that clinical widefield maFLIM endoscopy enables to image a plurality of autofluorescence metabolic and compositional biomarkers of oral epithelial dysplasia and cancer [20]. Our current findings indicate that six of these maFLIM-derived autofluorescence biomarkers are particularly relevant in the discrimination of dysplastic and cancerous vs. healthy oral tissue: $I_{390,n}$, $I_{452,n}$, $I_{390}/I_{500}$, $\tau_{avg,390}$, $\tau_{fast,452}$, and $\alpha_{fast,452}$ (Figure 2). Collagen in the lamina propria is the main contributor to the oral tissue autofluorescence induced by a 355 nm excitation wavelength and measured at the 390 ± 20 nm emission spectral band. Previous studies have reported lower normalized autofluorescence intensity measured at this band in cancerous and precancerous ($↓I_{390,n}$), relative to healthy, oral tissue associated to the breakdown of collagen crosslinks in the connective tissue [31,32] and increased epithelial thickness and tissue optical scattering, which are characteristic of premalignant and malignant oral tissue transformation [33]. We previously reported for the first time a faster average fluorescence lifetime measured at the 390 ± 20 nm emission spectral band in cancerous and dysplastic ($↓\tau_{avg,390})$ vs. healthy oral tissue [20]. Because of the spectral overlap of collagen and NADH at this band, this observation likely reflects a faster NADH autofluorescence temporal response signal resulting from decreased slower-decaying collagen signal in dysplastic and cancerous tissue. NADH within oral epithelial cells is the main contributor to the oral tissue autofluorescence induced by a 355 nm excitation wavelength and measured at the 452 ± 22.5 nm emission spectral band. Neoplastic cells are characterized by increased use of glycolysis in addition to oxidative phosphorylation [34], which reduces NAD+ into NADH, resulting in increased NADH/NAD+ ratio and quenched NADH autofluorescence [35]. This can be translated into increased normalized autofluorescence intensity ($↑I_{452,n}$) and shorter autofluorescence lifetime ($↓\tau_{fast,452}$, $↑\alpha_{fast,452}$) measured at the emission spectral band of 452 ± 22.5 nm in cancerous and dysplastic vs. healthy oral tissue. FAD within oral epithelial cells is the main contributor to the oral tissue autofluorescence induced by a 355 nm excitation wavelength and measured at the > 500 nm emission spectral band. Oxidative phosphorylation requires the oxidation of FADH_2_ into FAD, resulting in a higher concentration of mitochondrial FAD in neoplastic cells [35]. Hence, a decrease in the ratio of the autofluorescence intensities measured at the 390 ± 20 nm and > 500 nm spectral bands in cancerous and dysplastic oral lesions ($↓I_{390}/I_{500}$) compared to healthy oral tissue, which predominantly quantifies the contribution of collagen signal relative to that of FAD, could potentially represent a novel autofluorescence biomarker of oral cancer.

Several in vivo human studies have evaluated the potentials of autofluorescence spectroscopy or imaging for the discrimination of precancerous and cancerous from healthy oral tissue. De Veld et al. performed autofluorescence spectroscopy at six UV–vis excitation wavelengths in healthy volunteers (*n* = 95) and patients presenting either premalignant (*n* = 21) or malignant (*n* = 20) oral lesions [36]. Discrimination between healthy vs. cancerous oral lesions was performed with a Karhunen–Loeve linear classification model (KLLC) using features computed as ratios of intensities at specific emission wavelengths, and the performance was quantified in terms of ROC-AUC (> 0.9) following a leave-one-out cross-validation strategy. Discrimination between healthy and dysplastic lesions, on the other hand, was not achieved (ROC-AUC < 0.6). Kumar et al. performed autofluorescence spectroscopy at 405 nm excitation in healthy volunteers (*n* = 36) and patients presenting either premalignant (*n* = 38) or malignant (*n* = 67) oral lesions [37]. The data were divided into training and testing sets. Principal component analysis (PCA) was applied for feature extraction, and Mahalanobis-distance classification models were trained to discriminate autofluorescence spectra corresponding to either cancerous, precancerous, or healthy oral tissue. The classification performance was quantified in terms of sensitivity (70–100%) and specificity (86–100%). One limitation of this study was the use of multiple spectra per subject as independent datasets, resulting in not truly independent training and testing sets. Huang et al. performed autofluorescence imaging of NADH and FAD in healthy volunteers (*n* = 77) and patients presenting either premalignant (*n* = 34) or malignant (*n* = 49) oral lesions [38]. Different QDA classification models were trained to discriminate a manually selected region of interest (ROI) as either cancerous, precancerous, or healthy oral tissue using the ROI mean and standard deviation of the NADH and FAD emission intensities and their ratio as features. These models classified healthy vs. cancerous oral tissue ROIs with 94.6% sensitivity and 85.7% specificity, and healthy vs. precancerous oral tissue ROIs with 97.4% sensitivity and 38.2% specificity. One limitation of this study was the use of the same data for both training and validation. Jeng et al. performed autofluorescence imaging using the VELscope instrument (LED Dental, Vancouver-Canada) in healthy volunteers (*n* = 22) and patients presenting either premalignant (*n* = 31) or malignant (*n* = 16) oral lesions [39]. The data were divided into training and testing sets, the average and standard deviation of the autofluorescence intensity within ROIs were computed as features, and LDA and QDA models were trained for the discrimination among cancerous, precancerous, and healthy oral tissues. The classification performance was quantified in terms of ROC-AUC (0.8–0.97). One limitation of this study was the use of multiple (5) images per subject as independent datasets, resulting in not truly independent training and testing sets. Marsden et al. performed time-resolved autofluorescence spectroscopy (TRFS) in 53 patients undergoing upper aerodigestive oncologic surgery [40]. Different classification models were trained to discriminate an ROI as either cancerous or healthy oral tissue based on the classification of point-spectroscopy measurements taken within the ROI. The best leave-one-out cross-validation performance was obtained using a Random Forest model (ROC-AUC: 0.79–0.88). An independent validation performed on TRFS point measurements collected from nine patients excluded from the training stage resulted in significantly lower classification performance (ROC-AUC: 0.44–0.85). Discrimination between precancerous vs. healthy oral tissue was not achieved.

Our in vivo human study overcomes some limitations of the studies previously summarized. For positive surgical margin assessment, the discrimination of oral tissue in healthy volunteers from premalignant/malignant lesions in patients as performed in [36,37,38,39] is less relevant than the discrimination of healthy vs. premalignant/malignant oral tissue within the same patient as performed in [40] and our study. Point-spectroscopy measurements as performed in [36,37,40] are intrinsically slow and, thus, less suitable for surgical margin assessment than imaging approaches as in [38,39] and our study. The potential to discriminate precancerous from healthy oral tissue, as demonstrated in [37,39] and our study but not in [36,38,40], will be also relevant for surgical margin assessment. Finally, a very important difference of our study is the quantification of the classification performance in a totally independent testing set. The training and testing sets used in our study were collected at two different clinical centers in two different countries and using two different maFLIM endoscopic systems.

As previously discussed, automated detection of oral dysplasia and cancer based on autofluorescence spectroscopy and/or imaging can be attempted based on spectral/intensity and/or time-resolved autofluorescence features. Results from this study indicate that classification models trained with only spectral/intensity autofluorescence features can provide higher specificity but lower sensitivity than models trained with only time-resolved autofluorescence features, while ensemble classification models trained with both spectral/intensity and time-resolved features performed the best (Table 4, Figure 4). While detection based on only spectral/intensity autofluorescence features can be implemented with much simpler and significantly less costly instrumentation, our results indicate that time-resolved autofluorescence features can provide complementary discriminatory information. We recently reported a versatile and cost-efficient frequency-domain FLIM implementation that is being adopted in the design of novel multiwavelength-excitation and multispectral-emission FLIM endoscopic systems [41]; these novel instruments will further facilitate the clinical translation of maFLIM endoscopy.

The classification models explored in this study were limited to traditional ML models [24,25,26,27]. Even the optimal ML models identified during the training stage provided only modest (<80%) levels of specificity (Table 4). It is expected that with a more comprehensive training database and the adoption of more advanced ML models (e.g., deep learning methods) [42,43,44], it will be possible to enable automated discrimination of dysplastic and cancerous vs. healthy oral tissue with superior classification performance. Nevertheless, the classification results obtained in the independent maFLIM images used as testing set (ROC-AUC > 0.8, Figure 4) strongly support the potentials of an ML-enabled maFLIM-based strategy for automated and unbiased discrimination of dysplastic and cancerous vs. healthy oral tissue.

### Study Limitations

Although the independently validated results of this study clearly demonstrate the feasibility for ML-driven automated discrimination of dysplastic/cancerous from healthy oral tissue based on maFLIM endoscopy (ROC-AUC > 0.8), some study limitations were identified. The small sample size of both the training (*n* = 34) and testing (*n* = 23) sets used for developing and evaluating the performance of the CAD systems prevented the use of better performing state-of-the-art classification models. The demographics of the two different patient populations included in the training (Doha, Qatar) and testing (Dallas, Texas) sets (Table 2) could impact the classification performance of the ML models. The Qatar patient population mostly comprised Indian (35%) and Nepalese (21%) people with a 7.25:1 male-to-female ratio and an average age of 49 ± 11 years, while the Dallas patient population mostly comprised White people (77%) with a 1.09:1 male-to-female ratio and an average age of 62 ± 10 years. In addition, lesions imaged in Doha were clinically more advanced than those imaged in Dallas. These differences in race, gender, and age between patient populations and in malignancy stage could potentially affect the autofluorescence properties of the imaged oral tissues. Nevertheless, it was interesting to observe encouraging performance of a classification model that was trained with data from a particular patient/lesion population and independently tested on data from a distinct patient/lesion population. The lack of histopathology-based assessment of the maFLIM imaging data at the pixel-level and of images acquired specifically from lesion margins prevented to specifically quantify the capabilities of maFLIM endoscopy as a tool for discriminating negative vs. positive surgical margins. Finally, the current implementation of the optimized CAD systems did not allow for real-time processing of maFLIM data. Ongoing research efforts aiming to overcome these limitations include collecting maFLIM endoscopy images from both premalignant/malignant lesions and their visible margins, performing accurate pixel-level registration between the lesion maFLIM imaging data and histopathology tissue sections, and implementing optimized CAD systems using FPGA and GPU technologies for real-time maFLIM data processing, pixel-level classification, and tissue mapping visualization.

## 5. Conclusions

The results of this study further demonstrate the capabilities of maFLIM endoscopy to clinically image a plurality of metabolic and biochemical autofluorescence biomarkers of oral epithelial dysplasia and cancer. Moreover, these autofluorescence biomarkers were successfully used as features in machine-learning models optimized to discriminate dysplastic and cancerous from healthy oral tissue. Finally, this study demonstrates the first independent validation of a maFLIM endoscopy-based CAD system for automated clinical detection of dysplastic and cancerous oral lesions. Further developments in maFLIM instrumentation and image analysis methods could result in novel clinical tools for automated intraoperative image-guided in situ detection of positive margins during head and neck cancer resection surgery.

## Figures and Tables

**Table 1:** Distribution of the 57 imaged oral lesions based in both anatomical location and histopathological diagnosis (MiD: Mild Dysplasia; MoD: Moderate Dysplasia; HiD: High-Grade Dysplasia; SCC: Squamous Cell Carcinoma).

	Lesion Location		Histopathology Diagnosis			Total Number
Distribution of Imaged Oral Lesions		MiD	MoD	HiD	SCC	
Training Set	Buccal Mucosa	1	1	1	9	12
	Tongue	0	0	0	12	12
	Gingiva	0	0	2	3	5
	Lip	0	0	0	2	2
	Mandible	0	0	0	1	1
	Maxilla	0	0	0	1	1
	Floor of Mouth	0	0	0	1	1
	Total Number	1	1	3	29	34
Testing Set	Tongue	6	1	0	6	13
	Gingiva	1	0	0	5	6
	Buccal Mucosa	0	1	0	2	3
	Mandible	0	0	0	1	1
	Total Number	7	2	0	14	23

**Table 2:** Demographics of the two patient populations included in this study (MiD: Mild Dysplasia; MoD: Moderate Dysplasia; HiD: High-Grade Dysplasia; SCC: Squamous Cell Carcinoma).

Training Set (Doha, Qatar)					Testing Set (Dallas, Texas)				
Patient #	Race	Age	Gender	Histopathology	Patient #	Race	Age	Gender	Histopathology
1	Indian	34	M	SCC	1	White	59	M	SCC
2	Egyptian	67	M	SCC	2	White	76	F	SCC
3	Sri Lankan	52	M	SCC	3	White	N/A	F	SCC
4	Nepalese	47	M	SCC	4	Asian	N/A	F	SCC
5	Egyptian	42	M	SCC	5	White	60	M	SCC
6	Nepalese	35	M	HiD	6	White	N/A	M	MiD
7	Indian	50	M	HiD	7	White	54	F	MiD
8	Indian	51	M	SCC	8	White	75	F	MiD
9	Indian	43	M	MoD	9	Asian	58	M	MiD
10	Bangladeshi	59	M	SCC	10	Asian	N/A	M	MiD
11	Sri Lankan	55	M	MiD	11	White	55	F	MiD
12	Nepalese	31	M	SCC	12	White	N/A	M	MiD
13	Nepalese	39	M	SCC	13	White	N/A	M	MoD
14	Indian	36	M	SCC	14	White	62	F	SCC
15	Pakistani	36	M	SCC	15	White	59	M	SCC
16	Qatari	55	M	SCC	16	White	N/A	M	SCC
17	Indian	48	M	SCC	17	Asian	52	F	SCC
18	Nepalese	36	M	SCC	18	White	83	F	SCC
19	Indian	36	M	SCC	19	White	55	M	SCC
20	Pakistani	60	M	SCC	20	Black	N/A	F	MoD
21	Sudanese	61	F	SCC	21	White	N/A	M	SCC
22	Sudanese	60	F	SCC	22	White	68	M	SCC
23	Iranian	68	M	SCC	23	N/A	47	F	SCC
24	Indian	41	M	SCC					
25	Indian	49	M	SCC					
26	Nepalese	45	N/A	SCC					
27	Somali	60	M	SCC					
28	Indian	50	M	SCC					
29	Indian	61	M	SCC					
30	Indian	34	F	SCC					
31	Nepalese	30	M	HiD					
32	Filipino	49	F	SCC					
33	Iranian	59	M	SCC					
34	Pakistani	69	M	SCC					

**Table 3:** Summary of maFLIM-Derived Features Computed Per Pixel.

maFLIM Feature Category	Spectral Band			Total Number
	390 ± 20 nm	452 ± 22.5 nm	>500 nm	
Normalized Intensity	$I_{390,n}\left(x,y\right)$	$I_{452,n}\left(x,y\right)$	$I_{500,n}\left(x,y\right)$	3
Absolute Intensity Ratio	$I_{390}\left(x,y\right)$/$I_{452}\left(x,y\right)$			6
	$I_{390}\left(x,y\right)$/$I_{500}\left(x,y\right)$			
	$I_{452}\left(x,y\right)$/$I_{500}\left(x,y\right)$			
	$(I_{452}\left(x,y\right)+I_{500}\left(x,y\right))/I_{390}\left(x,y\right)$			
	$(I_{390}\left(x,y\right)+I_{500}\left(x,y\right))/I_{452}\left(x,y\right)$			
	$(I_{390}\left(x,y\right)+I_{452}\left(x,y\right))/I_{500}\left(x,y\right)$			
Time-Resolved	$\tau_{fast,390}\left(x,y\right)$	$\tau_{fast,452}\left(x,y\right)$	$\tau_{fast,500}\left(x,y\right)$	12
	$\tau_{slow,390}\left(x,y\right)$	$\tau_{slow,452}\left(x,y\right)$	$\tau_{slow,500}\left(x,y\right)$	
	$\alpha_{fast,390}\left(x,y\right)$	$\alpha_{fast,452}\left(x,y\right)$	$\alpha_{fast,500}\left(x,y\right)$	
	$\tau_{avg,390}\left(x,y\right)$	$\tau_{avg,452}\left(x,y\right)$	$\tau_{avg,500}\left(x,y\right)$	
Total Number				21

**Table 4:** Cross-Validation Classification Performance On the Training Set For Each maFLIM Feature Pool and Classification model.

maFLIM Feature Pool	Classification Model	F1-Score	Sensitivity	Specificity
Spectral	LDA	0.78	82%	71%
	QDA	0.74	76%	71%
	**SVM**	**0.79**	**82%**	**74%**
	LOGREG	0.79	85%	71%
Time-Resolved	LDA	0.75	79%	68%
	**QDA**	**0.83**	**91%**	**71%**
	SVM	0.73	76%	68%
	LOGREG	0.76	79%	71%
Top three Spectral	SVM	0.76	79%	71%
Top three Time-Resolved	QDA	0.82	91%	68%
Ensemble (Top three Spectral and Time-Resolved)	**SVM-QDA**	**0.85**	**94%**	**74%**

**Table 5:** Confusion matrices from the 7-fold cross-validation using the optimal model for each maFLIM feature pool (MiD: Mild Dysplasia; MoD: Moderate Dysplasia; HiD: High-Grade Dysplasia, SCC: Squamous Cell Carcinoma).

Confusion Matrices for Best Performing Models		Predicted					
		SVM (Spectral)		QDA (Time-Resolved)		SVM-QDA (Ensemble)	
		(−)	(+)	(−)	(+)	(−)	(+)
**True**	Healthy (*n* = 34)	25	9	24	10	25	9
	MiD (*n* = 1)	1	0	0	1	0	1
	MoD (*n* = 1)	0	1	0	1	0	1
	HiD (*n* = 3)	1	2	0	3	0	3
	SCC (*n* = 29)	4	25	3	26	2	27
	Total	31	37	27	41	27	41

## Data Availability

The data presented in this study are available on request from the corresponding author.

## Supplementary Materials

The following are available online at https://www.mdpi.com/article/10.3390/cancers13194751/s1, Figure S1: Posterior probability maps of 23 dysplastic/cancerous oral lesions and paired healthy oral tissues obtained from the SVM-QDA ensemble classifier, Table S1: Confusion matrices resulting from the application of the optimal classification models to the testing set.
