# Supplementary material for: Machine-Learning Assisted Discrimination of Precancerous and Cancerous from Healthy Oral Tissue Based on Multispectral Autofluorescence Lifetime Imaging Endoscopy
Source: Cancers (Basel). 2021 Sep 23;13(19):4751. doi: 10.3390/cancers13194751 (PMC8507537; doi:10.3390/cancers13194751)
Supplement: Supplementary file 1 [file cancers-13-04751-s001.zip › cancers-1293841-supplementary.pdf]

Supplemental Material

Figure S1 shows the posterior probability maps produced by the SVM-QDA ensemble classification model (red intensity scale) superposed on the total fluorescence intensity maps (grey intensity scale) for all maFLIM images in the testing set.

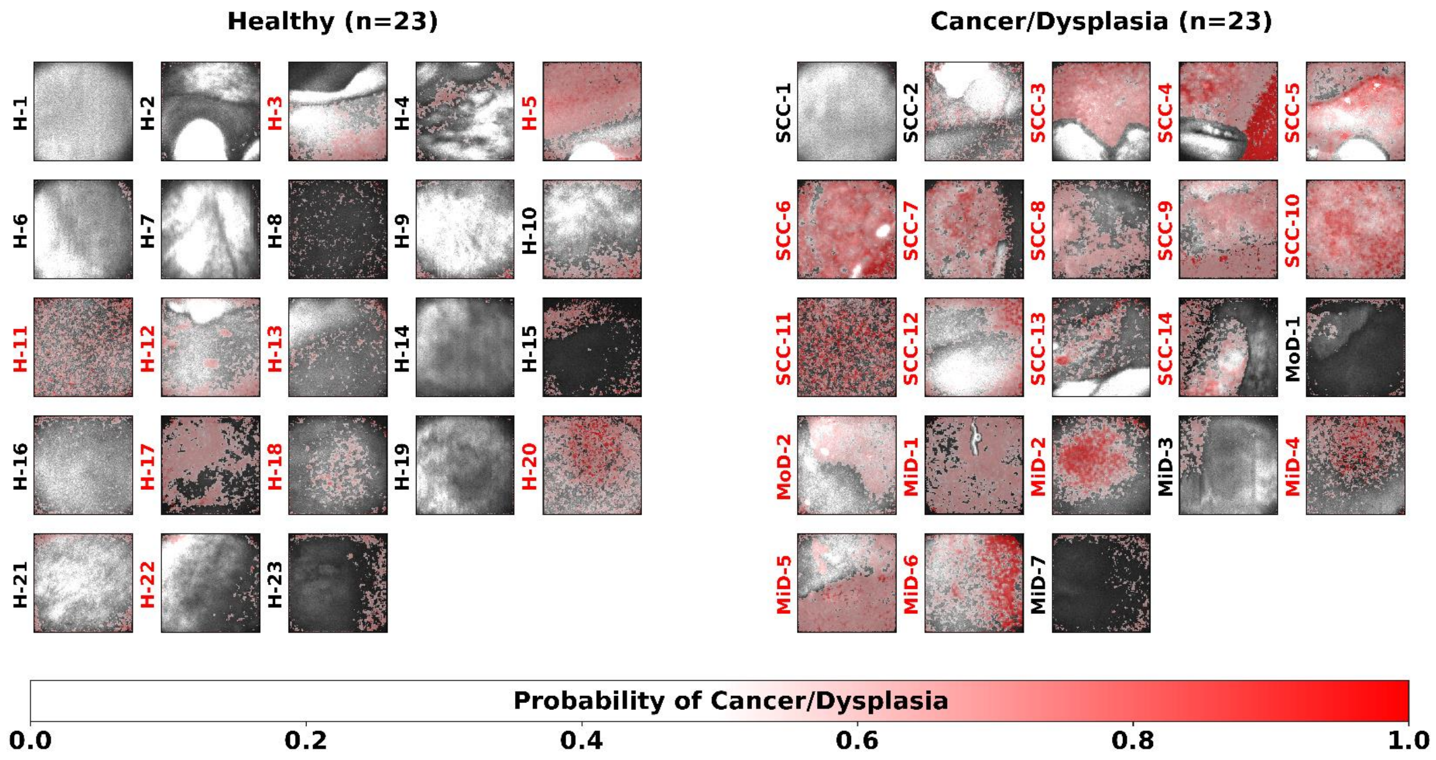

Figure. S1. Posterior probability maps (red intensity scale) superposed on the total fluorescence intensity maps (grey intensity scale) of 23 dysplastic/cancerous oral lesions and paired healthy oral tissues obtained from the SVM-QDA ensemble classifier. Sample identification numbers are shown on the left of each map and color-coded in red if classified positive according to the predicted image-level classification.

Table S1 presents the confusion matrices resulting from the application of the optimal classification models to the testing set**.** The sensitivity, specificity, and F-1 score were computed after applying an image-level score threshold optimized on the training set by selecting the point on the ROC curve with maximum sensitivity within the (1-specificity) range of 0%-30%. The images in the testing set were classified as positive (dysplasia/cancer) if the image-level scores were greater than or equal to the threshold obtained from the training set, or as negative (healthy) otherwise.

The spectral-only SVM model achieved very high specificity (87%), but very low sensitivity (22%) and F1-score of 0.32, while the time-resolved-only QDA model displayed higher sensitivity (74%) but lower specificity (52%) and F1-score of 0.67. The SVM-QDA ensemble model achieved more balanced levels of sensitivity (78%), specificity (61%), and F1-score (0.72). Noticeable, 12 out of 14 (>85%) of SCC lesions were correctly classified.

Table S1. Confusion matrices resulting from the application of the optimal classification models to the testing set (**MiD:** Mild Dysplasia; **MoD:** Moderate Dysplasia; **SCC:** Squamous Cell Carcinoma)

		**Predicted**					
		**SVM (Spectral)**		**QDA (Time-Resolved)**		**SVM-QDA (Ensemble)**	
		(−)	(+)	(−)	(+)	(−)	(+)
**True**	**Healthy (n=23)**	20	3	12	11	14	9
	**MiD (n=7)**	5	2	3	4	2	5
	**MoD (n=2)**	2	0	1	1	1	1
	**SCC (n=14)**	11	3	2	12	2	12
	**Total**	38	8	18	28	19	27
